# Angiotensins Inhibit Cell Growth in GH3 Lactosomatotroph Pituitary Tumor Cell Culture: A Possible Involvement of the p44/42 and p38 MAPK Pathways

**DOI:** 10.1100/2012/189290

**Published:** 2012-04-24

**Authors:** Dorota Ptasinska-Wnuk, Hanna Lawnicka, Slawomir Mucha, Jolanta Kunert-Radek, Marek Pawlikowski, Henryk Stepien

**Affiliations:** ^1^Department of Endocrinology, The County Hospital of Kutno, 52 Kosciuszki Street, 99-300 Kutno, Poland; ^2^Department of Immunoendocrinology, Chair of Endocrinology, Medical University of Lodz, Dr. Sterling 3 Street, 91-425 Lodz, Poland; ^3^Clinic of Endocrinology, Medical University of Lodz, Dr. Sterling 3 Street, 91-425 Lodz, Poland

## Abstract

The local renin-angiotensin system is present in the pituitary. We investigated the effects of angiotensins on GH3 lactosomatotroph cells proliferation in vitro and the involvement of p44/42 and p38 MAPK inhibitors in the growth-regulatory effects of angiotensins. *Materials and Methods*. Cell viability using the Mosmann method and proliferation by the measurement of BrdU incorporation during DNA synthesis were estimated. *Results*. Ang II and ang IV decreased the viability and proliferation of GH3 cells. Inhibitor of p44/42 MAPK attenuated the effects of ang II on cell viability and proliferation but did not affect the ang 5-8-dependent actions. Inhibitor of p38 MAPK prevented the decrease in the number of GH3 cells in ang-II- and ang-IV-treated groups. *Conclusions*. The growth-inhibitory effect of ang II is possibly mediated by the p44/42 MAPK. The p38 MAPK appears to mediate the inhibitory effects of both ang II and ang 5–8 upon cell survival.

## 1. Introduction

The existence of intrinsic tissue angiotensin-generating bioenzymatic cascades, independent of the classical circulating renin-angiotensin system (RAS), has been established in several organs including heart [1, 2], blood vessels [3, 4], brain [5], kidney [6, 7], endocrine system (adrenals, thymus, ovary, testis, prostate) [8], gastrointestinal tract [9], liver, pancreas [10], as well as adipose and hematopoietic tissues (reviewed in details by Leung [11]). Angiotensin II (ang II), the main bioactive peptide of RAS, is cleaved by aminopeptidase A (APA) into a shorter peptide ang III (ang 2–8) [12]. Aminopeptidase N (APN) is, in turn, responsible for the conversion of ang III into ang IV (ang 3–8) [13, 14]. The subsequent degradation of ang IV leads to the formation of ang 4–8 and ang 5–8. Locally synthesized ang II has been defined to act as an autocrine and paracrine growth factor in many tissues [15] including vascular smooth muscle [16], heart muscle [17], adrenal cortex [18, 19], renal mesangium [20] and epithelium [21], or prostatic stroma [22]. Moreover, the increasing lines of evidence indicate the additional effects of ang II derivatives on cellular growth. Ang III and ang IV have been demonstrated to inhibit the growth of the androgen-independent human prostate cancer line in vitro [23]. Ang IV was additionally shown to induce neurite outgrowth and to stimulate proliferation in the uterine endometrium [24], adrenal zona glomerulosa [25], prostatic epithelium [26], and vascular endothelium [27]. Few attempts have been undertaken to understand the physiological functions of ang 4–8 or ang 5–8, and most authors consider the ang IV derivatives to be biologically inactive.

Angiotensin peptides act via at least three angiotensin receptors, designated AT1, AT2, and AT4. Ang IV binds preferentially to the specific receptor subtype called AT4, which shows poor affinity to ang IV precursors. Besides an intracellular signal transduction mechanisms characteristic for G-protein-coupled receptors, angiotensins are able to modulate the activity of the tyrosine-kinase-receptor- (TKR-) linked pathways, including mitogen-activated protein kinases (MAPKs) [28–33]. MAPKs belong to the family of protein serine/threonine kinases, and they have been divided into three main groups: the extracellular-regulated kinases 1/2 (ERK1/2 or MAPK p44/42), MAP p38, and the c-jun-N-terminal kinases (JNK). ERK1/2 is typically activated by the growth factors and cytokines via TKR and its downstream effectors following Ras, Raf, and mitogen-activated protein kinase kinase (MAPKK or MEK) [34, 35]. This group is known to mediate mitogenic and antiapoptotic effects. MAP p38, and JNK respond to the proinflammatory cytokines, cellular stress, heat shock, and hyperosmolarity and are usually connected with antiproliferation and promotion of apoptosis [36, 37]. The well-defined effects of angiotensin peptides via MAPKs imply the role of RAS in the pathogenesis of various disorders, such as vascular hypertrophy and remodeling or hypertrophy of renal proximal tubular cells leading to the tubulointerstitial fibrosis [38–40]. Ang II has been also suggested to affect the development of some neoplasms including acute myeloid leukemia, prostate cancer, pancreatic cancer, or C6 glioma of rat [41–44].

The existence of an intrinsic angiotensin-generating system in the anterior pituitary has been provided by recent immunohistochemical and molecular studies [45–47]. They have shown that the components of RAS including peptides, receptors, and enzymes are present in all types of the hormone-secreting anterior pituitary cells, whereas the specific-binding sites for ang II are localized mainly on lactotropes. Locally produced ang II is known to affect the release of anterior pituitary hormones in a paracrine and autocrine manner. This peptide is known to promote secretion of prolactin (PRL) and adrenocorticotropic hormone (ACTH) and, to a lesser extent, growth hormone (GH) and luteinizing hormone (LH) [48]. Earlier in vitro and in vivo studies have also suggested the involvement of RAS in regulation of lactotropes proliferation and the engagement of this system in pathogenesis of estrogen-induced PRL-secreting adenoma [49]. Nevertheless, there are no reports on the postreceptor mechanism connected with angiotensin action on the lactotropic cell growth. In the present study, we employed lactosomatotroph GH3 cells in order to determine the influences of ang II and its derivatives, ang IV, and ang 5–8, on anterior pituitary tumor cell proliferation. Using the inhibitors of two MAPK cascades, we also examined the potential association of MAPK p44/42 and MAPK p38 with effects of angiotensin peptides. 

## 2. Materials and Methods

### 2.1. Materials

The chemicals used in present experiments were obtained from the following sources: angiotensin II (ang II,* Sigma*), angiotensin IV (ang IV, *Bachem*), angiotensin 5–8 (ang 5–8, Bachem), PD98059 (*Sigma*), SB203580 (*Sigma*), dimethyl sulfoxid, DMSO, C2H6OS (*Merck*), nutrient mixture F-10 (Ham) (*Sigma*), sodium bicarbonate, NaHCO_3_ (*Sigma*), penicillin/streptomycin solution (*Sigma*), fetal bovine serum, FBS (Sigma), horse serum (*Sigma*), trypsin-EDTA (*Sigma*). 

### 2.2. Cell Culture

The rat lactosomatotroph tumor cell line GH3 (CCL-82.1) was obtained from *ATCC, LGC Promochem*. GH3 cells are a clonal strain of rat pituitary tumor cells. They have many properties common to both normal lactotropes and lactosomatotropes, being thus valuable model for the investigations of functions of PRL-secreting cells. Continuous GH3 cell culture was maintained in culture flasks (Nunc Eas Y Flask 25 cm^2^, NUNC). All the cells were grown in Ham's F-10 medium supplemented with 1.2 g/L sodium bicarbonate, 100 U/mL penicillin and 100 *μ*g/mL streptomycin solution, 15% heat-inactivated horse serum and 2.5% heat-inactivated fetal bovine serum at 37°C and in a humidified atmosphere of 95% air and 5% CO_2_. Every 7 days, the cells were harvested after 2 min incubation at 37°C in the presence of trypsin-EDTA (0.05 or 0.02%, resp.) in Hanks balanced solution. The cells were washed twice in complete F-10 medium and, after the last centrifugation seeded at 1 × 10^5^ cells in 5 mL of fresh medium. 

### 2.3. Cell Viability Study

 The cells were subjected to the trypsinization process and then suspended at 4 × 10^5^/mL in complete F-10 medium. 50 *μ*L aliquots of cell suspension (2 × 10^4^ cells) were placed in the wells of plastic 96-well culture plates (96 Cell Culture Cluster Dish, Costar; Nunclon, Microwell Plates, *NUNC*). After 12 hr period of preincubation (5% CO_2_, 37°C, 95% humidity), the tested substances were added to the appropriate wells: in experiment (1), ang II or ang IV at final concentrations of 10^−6^ M, 10^−8^ M, 10^−10^ M, or 10^−12^ M; 10^−8^ M ang II or 10^−8^ M ang IV + amastatin at final concentrations of 10^−5^ M, 10^−6^ M, or 10^−7^ M. The appropriate volume of the culture medium was added to the wells of control group and to the wells with one tested substance to the final volume 200 *μ*L in each well. In experiment (2), ang II at final concentration of 10^−8^ M, ang 5–8 at final concentration of 10^−10^ M, PD98059 at final concentration of 10^−5 ^M, SB203580 at final concentration of 10^−5^ M; 10^−8^ M ang II or 10^−10^ M ang 5–8 + 10^−5^ M PD98059; 10^−8^ M ang II or 10^−10^ M ang 5–8 + 10^−5^ M SB203580. Both PD98059 and SB203580 were dissolved in the DMSO/medium solution, in the proportion DMSO : medium 37 : 63 for PD98059 and 1 : 9 for SB98059. Since DMSO is known to affect cellular viability, the appropriate solution of culture medium with DMSO was added to the wells of control group and to the wells with ang II or ang 5–8 alone, to obtain the final DMSO concentration equivalent to that in the PD98059- or SB203580-treated groups. Finally, the appropriate volume of the culture medium was added to the wells of control group and to the wells with one tested substance, to the final volume 200 *μ*L in each well.

After 72 hrs of incubation (5% CO_2_, 37°C, 95% humidity), cell viability was estimated using the modified Mosmann method, following the procedure recommended by the producer of the kit (EZ4Y, Easy for You, the 4th Generation Non Radioactive Cell Proliferation and Cytotoxicity Assay, Biomedica Gruppe, Austria, *Bellco Biomedica Poland*). The optical density (OD) of each sample was measured by a microplate reader at 450 nm.

### 2.4. Cell Proliferation Study

 After trypsinization process, the cells were suspended at 3 × 10^5^/mL in complete F-10 medium. 50 *μ*L aliquots of cell suspension (1.5 × 10^4^ cells) were placed in the wells of plastic 96-well culture plates (96 Cell Culture Cluster Dish, Costar; Nunclon, Microwell Plates, NUNC). After 12 hrs period of preincubation (5% CO_2_, 37°C, 95% humidity), the tested substances were added to the appropriate wells: in experiment (1), ang II, ang IV, or ang 5–8 at final concentrations of 10^−12^ M, 10^−10^ M, 10^−8^ M, or 10^−6^ M. The appropriate volume of the culture medium was added to the wells of control group the final volume 100 *μ*L in each well. In experiment (2), ang II at final concentration of 10^−8^ M, ang 5–8 at final concentration of 10^−10^ M, PD98059 at final concentration of 10^−5^ M, SB203580 at final concentration of 10^−5^ M; 10^−8^ M ang II or 10^−10^ M ang 5–8 + 10^−5^ M PD98059; 10^−8^ M ang II or 10^−10^ M ang 5–8 + 10^−5^ M SB203580. Both PD98059 and SB203580 were dissolved in the DMSO/medium solution, in the proportion DMSO : medium 37 : 63 for PD98059 and 1 : 9 for SB98059. Since DMSO is known to affect cellular proliferation, the appropriate solution of culture medium with DMSO was added to the wells of control group and to the wells with ang II or ang 5–8 alone, to obtain the final DMSO concentration equivalent to that in the PD98059- or SB203580-treated groups. Finally, the appropriate volume of the culture medium was added to the wells of control group and to the wells with one tested substance, to the final volume 100 *μ*L in each well.

 The cells were cultured in the presence of the tested substances for 72 hrs (5% CO_2_, 37°C, 95% humidity). After 68 hrs of incubation, 10 *μ*L BrdU labeling solution (final concentration: 10 *μ*M BrdU) was added to each well, and the cells were reincubated for additional 4 hrs, necessary for pyrimidine analogue BrdU to incorporate into DNA of proliferating cells. Then, cell proliferation was measured following the procedure recommended by the producer of the kit (Cell Proliferation ELISA, BrdU (colorimetric), colorimetric immunoassay for the quantification of cell proliferation, based on the measurement of BrdU incorporation during DNA synthesis, a nonradioactive alternative to the [^3^H]-thymidine incorporation assay, *Roche*). The optical density (OD) of each sample was measured by a microplate reader at 450 nm.

### 2.5. Statistical Evaluation

 Data are given as the absolute values of extinction and as the percentage of the optical density (OD) measured at 450 nm of unstimulated cells (= 100%) and represents 8–10 different measurements ± SD. The normality of distribution of the results was examined by the Student test. Comparisons of individual groups were evaluated by analysis of variance (ANOVA). Differences were considered significant if *P* < 0.05.

## 3. Results

**Figure 1:**
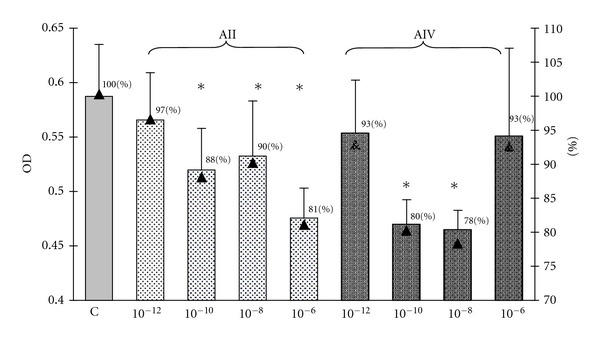
The influence of 72 hr treatment with angiotensin II (AII) and angiotensin IV (AIV) on the cellular viability in the lactosomatotroph GH3 cell culture. *X* axis: absolute values of the optical density (OD), auxiliary axis (▲): OD in the particular angiotensin-treated groups expressed as the percentage of the optical density measured at 450 nm of unstimulated cells (control (C) = 100%). *X* ± SEM; *P < 0.05 versus C.

**Figure 2:**
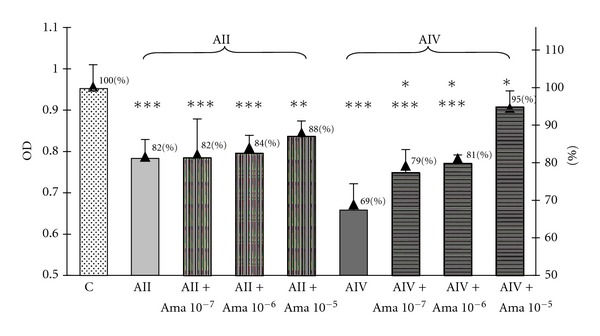
The influence of aminopeptidases inhibitor amastatin (Ama) at concentrations 10^−7^ M, 10^−6^ M, and 10^−5^ M on angiotensin II (AII)- and angiotensin IV (AIV)-induced decrease of the cellular viability in the lactosomatotroph GH3 cell culture. *X* axis: absolute values of the optical density (OD), auxiliary axis (▲)—OD in the particular groups expressed as the percentage of the optical density measured at 450 nm of unstimulated cells (control (C) = 100%). *X* ± SEM; ***P < 0,001 versus C, **P < 0,01 versus C, *P < 0,05 versus AIV.

**Figure 3:**
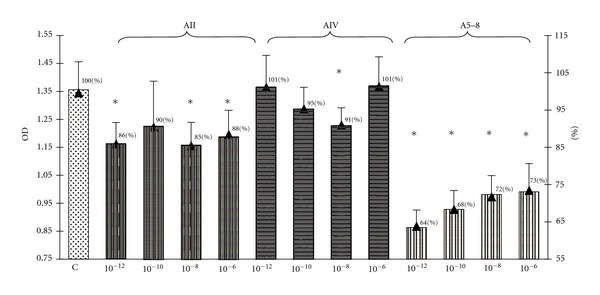
The influence of 72-hrs treatment with angiotensin II (AII), angiotensin IV (AIV), and angiotensin 5–8 (A5–8) on the cellular proliferation expressed as BrdU incorporation in the lactosomatotroph GH3 cell culture. *X* axis: absolute values of the optical density (OD), auxiliary axis (▲): OD in the particular angiotensin-treated groups expressed as the percentage of the optical density measured at 450 nm of unstimulated cells (control (C) = 100%). *X* ± SEM; *P < 0.05 versus C.

As demonstrated in Figure 1, the treatment of GH3 cells with ang II at concentrations of 10^−6^ M, 10^−8^ M, 10^−10^ M and with ang IV at concentrations 10^−8^ M and 10^−10^ M resulted in an inhibition of the total culture viability. Since culture viability has been proved as parallel to the number of metabolically active cells, these data imply the examined angiotensin peptides decrease the number of GH3 cells in culture. Amastatin, the inhibitor of aminopeptidases A and M, abolished—partially at concentrations of 10^−6^ M–10^−7^ M and completely at concentration of 10^−5^ M—the inhibitory effect of ang IV (Figure 2). In contrast, pretreatment with amastatin did not prevent the decrease in the number of GH3 cells in response to ang II (Figure 2). Determination of the cellular proliferation using BrdU incorporation method revealed that ang II at concentrations 10^−6^ M, 10^−8^ M, 10^−12^ M, and ang IV at concentration 10^−8^ M decreased also BrdU uptake in GH3 culture (Figure 3). Antiproliferative effect has been additionally shown with respect to the ang IV degradation product, ang 5–8 (Figure 3).

**Figure 4:**
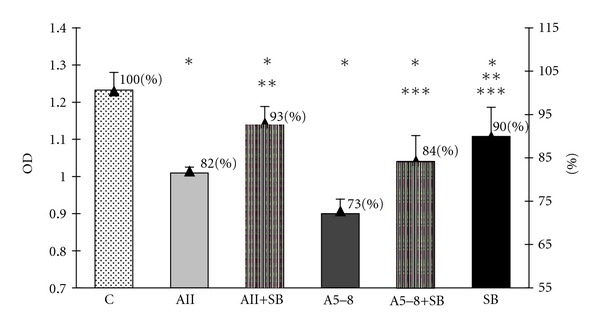
The influence of p38 MAPK inhibitor SB203580 at concentration 10^−5^ M on angiotensin-II-(AII-) and angiotensin-5–8-(A5–8-) induced decrease of the cellular viability in the lactosomatotroph GH3 cell culture. *X* axis: absolute values of the optical density (OD), auxiliary axis (▲): OD in the particular groups expressed as the percentage of the optical density measured at 450 nm of unstimulated cells (control (C) = 100%). *X* ± SEM; *P < 0.05 versus C, **P < 0.05 versus AII, ***P < 0.05 versus A5–8.

**Figure 5:**
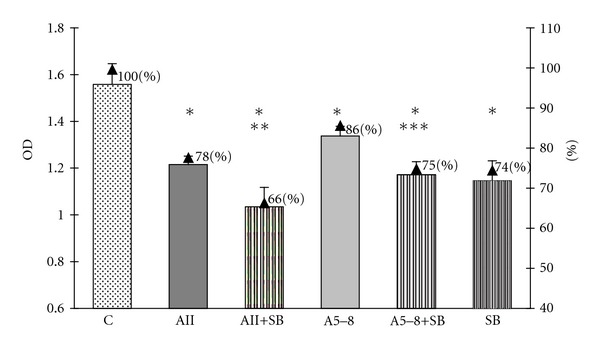
The influence of p38 MAPK inhibitor SB203580 at concentration 10^−5^ M on angiotensin-II-(AII-) and angiotensin-5–8-(A5–8-) induced decrease of the BrdU incorporation into lactosomatotroph GH3 cells. *X* axis: absolute values of the optical density (OD), auxiliary axis (▲): OD in the particular groups expressed as the percentage of the optical density measured at 450 nm of unstimulated cells (control (C) = 100%). *X* ± SEM; *P < 0.05 versus C, **P < 0.05 versus AII, ***P < 0.05 versus A5–8.

**Figure 6:**
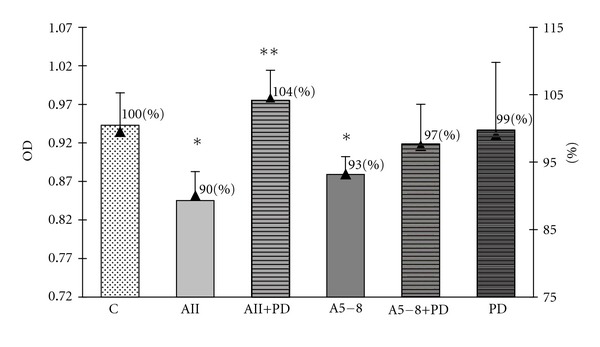
The influence of p44/42 MAPK inhibitor PD98059 at concentration 10^−5^ M on angiotensin-II-(AII-) and angiotensin-5–8-(A5-8-) induced decrease of the cellular viability in the lactosomatotroph GH3 cell culture. *X* axis: absolute values of the optical density (OD), auxiliary axis (▲): OD in the particular groups expressed as the percentage of the optical density measured at 450 nm of unstimulated cells (control (C) = 100%). *X* ± SEM; *P < 0.05 versus C, **P < 0.05 versus AII.

**Figure 7:**
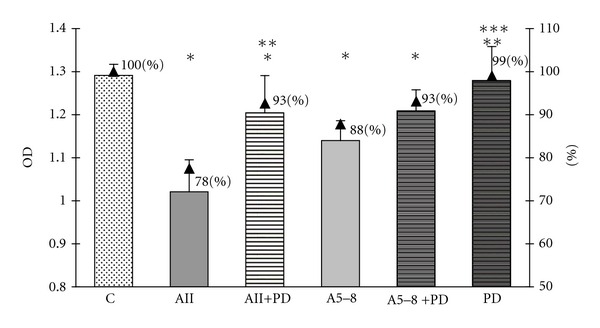
The influence of p44/42 MAPK inhibitor PD98059 at concentration 10^−5^ M on angiotensin-II-(AII-) and angiotensin-5–8-(A5–8-) induced decrease of the BrdU incorporation into lactosomatotroph GH3 cells. *X* axis: absolute values of the optical density (OD), auxiliary axis (▲): OD in the particular groups expressed as the percentage of the optical density measured at 450 nm of unstimulated cells (control (C) = 100%). *X* ± SEM; *P < 0.05 versus C, **P < 0.05 versus AII, ***P < 0.05 versus A5–8.

In order to examine an involvement of two MAPK pathways, the p44/42 MAPK and p38 MAPK, in the observed effects of angiotensin peptides in GH3 cell culture, we used the specific inhibitor of MEK phosphorylation PD98059 and the specific inhibitor of p38 MAPK SB203580. Both inhibitors were used at concentrations of 10 *μ*M (10^−5^ M), the level at which they have been proved to block the activity of MAPK p44/42 and MAPK p38. We revealed that PD98059 attenuated the inhibitory effects of ang II on both cell viability and proliferation but did not affect the ang-5–8-dependent actions (Figures 6 and 7). SB203580 partially prevented the decrease in the number of GH3 cells in both ang-II- and ang-IV-treated groups (Figure 4) but did not impair the antiproliferative effects of the peptides (Figure 5). Moreover, p38 MAPK inhibitor alone lowered basal number and proliferation of GH3 cells (Figures 4 and 5).

## 4. Discussion

Numerous cytokines, growth factors, and hormones have been found to be implicated in the pituitary tumor development. Phosphorylation of the MAP kinases via the receptors with intrinsic tyrosine kinase activity has been defined as essential to the growth-regulatory effects of many factors. Some of the TKRs may be coexpressed with their ligands, thus forming the intrapituitary autocrine loops that stimulate adenoma cell growth. The pituitary RAS is possibly one of these regulatory loops, as the components of this system including precursors, enzymes, and the receptors have been identified within the cells of various anterior pituitary tumors as well as in the cells of lactosomatotrop GH3 line. On the other hand, the involvement of angiotensin peptides in the control of cellular proliferation within anterior pituitary has previously been demonstrated. 

 We found that all angiotensin peptides investigated, ang II, ang IV, and ang 5–8, inhibited cellular proliferation, and decreased the number of viable cells in GH3 cell culture. Our study is not the first to demonstrate antiproliferative effects of angiotensins. Ang II has been found to decrease the cell growth in primary culture of rat adrenal glomerulosa cells and the bFGF-induced proliferation of bovine adrenal fasciculate cells [50, 51]. Ławnicka et al. revealed the inhibitory actions of ang II, ang III, and ang IV on the growth of DU-145 human prostate cancer cell line in vitro [23]. On the other hand, the results of the present study seem to stay in opposition with the earlier data, showing the stimulatory effect of ang II and ang IV on normal pituitary lactotrophs or estrogen induced prolactinomas either in vivo and in vitro [49, 52–54]. The discrepancy between our results and the results of previous experiments may be possibly explained by the application of different experimental models. First, the estrogen-induced-prolactinoma cells and lactosomatotroph GH3 cells, in spite of many similarities, represent the distinct cell types certainly differing at the genomic level. Second, under in vivo conditions, the growth-promoting effects of angiotensins may be mediated by their involvement in the control of angiogenesis.

 In GH3 cell culture, the inhibitory effect of ang IV on the cell viability is completely abolished by amastatin, whereas this inhibitor of aminopeptidases did not influence the ang-II-induced effect. The latter finding suggests that growth-inhibitory action of ang II in GH3 cell culture does not depend on its conversion into smaller fragments. With respect to ang IV, the observed abolition of its effects by amastatin is rather unexpected. Amastatin has been demonstrated to inactivate selectively APA and APN, the enzymes responsible for the N-terminal degradation of ang II and ang III, respectively [55–57]. However, our results suggest that the action of this aminopeptidases inhibitor may be extended on the inactivation of other than APA/APN enzymes, including ang IV degrading peptidases. Presuming such an activity of amastatin, we would hypothesize that growth-inhibitory effect of ang IV in GH3 cell culture is determined by its previous conversion into the smallest peptides. Such a mechanism is highly probable in the context of the reported above antiproliferative effect of ang 5–8 in the culture of GH3 cells. For our best knowledge, the present study is the first one demonstrating the ability of ang 5–8 to regulate cellular proliferation and the possible action of ang IV via its degradation products.

Phosphorylation of the MAP kinases via the receptors with intrinsic tyrosine kinase activity has been defined as essential to the growth-regulatory effects of many factors. The earlier papers from our laboratory showed the stimulation of global TRK activity in rat anterior pituitary glands and the estrogen-induced rat pituitary prolactinomas by ang II and ang IV [52, 53] and by ang III and ang 1–7 as well [58, 59]. The present study revealed that the inhibitor of the p44/42 MAPK pathway, PD98059, abolished the inhibitory effects of ang II on both BrdU incorporation and total cell viability, but did not affect the ang 5–8-dependent actions. These data suggest that, in GH3 cell culture, ERK phosphorylation may be essential for the ang-II-induced antiproliferation. In contrast to ang II, ang 5–8 appears to decrease proliferation and total viability of the GH3 cells independently on the MEK-ERK phosphorylation. Nevertheless, our results do not exclude regulatory influence of ang 5–8 upon the p44/42 MAPK activity via other mechanisms, that is, MAPK phosphatases (MKP) inactivation.

An involvement of p44/42 MAPK in the antiproliferative effects may seem surprising from the point of view of earlier studies. The activation of ERK1/2 was critical for the growth-promoting actions of ang II in cardiac fibroblasts or prostate cancer cells in cultures [41]. These MAPKs were also demonstrated to participate in the ang-IV-dependent mitogenic effects in cultured lung endothelial cells [27]. Similar results have been obtained with respect to the lactotroph and lactosomatotroph cells. ERK1/2 were shown to mediate the bFGF-induced proliferation in PRL lactotroph cells and enriched lactotropes [60]. Furthermore, the inhibitory effect of somatostatin on the GH3 cell cycle progression seemed to be dependent on the decrease in MAPK activity [61, 62]. However, there are also reports on the association of ERK cascade with antiproliferative effects. In GH3 cell culture, both thyrotropin-releasing hormone (TRH) and epidermal growth factor (EGF) induced ERK activity that correlated with reduced DNA synthesis and concomitant differentiation of lactosomatotropes into lactotropes [63, 64]. Otis et al. [50] revealed that ang-II-stimulated MAPK p44/42 phosphorylation led to an increase in p27^Kip1^ expression and to the subsequent inhibition of cellular proliferation in cultured rat glomerulosa cells. Later studies indicated that ang-II-induced inhibition of proliferation of rat adrenal glomerulosa cells involved both p42/p44 MAPK and p38 MAPK activation [65]. The identical mechanism of action was responsible for ang-II-mediated inhibition of cell cycle progression in renal proximal tubular cells [40]. At the end, ang-II-induced ERK activity caused the hypertrophy of vascular smooth muscle cells (VSMCs), as opposed to the platelet-derived growth-factor-(PDGF-) dependent cell proliferation [66].

The whole of former studies suggest that the same intracellular pathways may be shared between proliferation and differentiation [67]. It has been demonstrated that mitogenic effects usually appeared in response to the transient ERK activity, whereas the sustained MAPK induction led to antiproliferative responses [68–71]. In the view of these results, we might speculate that strong persistent activation of an unidentified receptor by ang II may result in the sustained ERK activation and subsequent inhibition of cellular proliferation in GH3 cell culture. Furthermore, in context of previously reported mitogenic properties of ERK-dependent signals in GH3 cells, our results strengthen the hypothesis that regulation of the cellular growth and by MAPK is possibly cell specific and dependent on the stimulus [72].

As reported above, inhibitor of p38 MAPK blunted the ang-II- and ang-5–8-induced decrease in number of the viable GH3 cells. In contrast, SB203580 did not affect the inhibitory effects of angiotensin peptides on BrdU incorporation. Our study clearly indicates that Mosmann's and BrdU incorporation techniques do not provide the parallel results. Indeed, it should be mentioned, that Mosmann's method estimates the total number of viable cells, but does not discriminate between the effects depending on the changes in proliferation, necrosis or apoptosis. In contrast, BrdU-incorporation serves as a direct mitotic index, and it reflects the changes concerning only the cellular proliferation. All these findings suggest that p38 MAPK mediates neither the antiproliferative effects of ang II nor ang 5–8, but it is possibly connected with inhibitory effects of both peptides upon the cell survival, that is, via the induction of apoptosis. This presumption is highly probable in the context of previously reported association of the p38 MAPK pathway with apoptosis [73, 74]. Furthermore, bromocriptine-induced p38 MAPK activation was implicated in apoptosis of the bromocriptine-treated GH3 cells [75].

Inhibitory effect of SB203580 on the basal cell number and proliferation is intriguing. Nevertheless, it is worth recalling that inhibitors of p38 MAPK are known to block arachidonic acid metabolism in several cell types [72, 76]. On the other hand, one of the arachidonic acid derivatives, 12-lipooxygenase product 12-hydroxyeikosatetraenoic acid, has been found to increase cellular proliferation [77]. There is also possibility of the direct cytotoxic action of SB203580 on GH3 cells.

Concluding our study, ang II, ang IV, and ang 5–8 decrease the cell number and proliferation in GH3 cell culture. The growth-inhibitory effects seem to be dependent on the direct interaction of ang II with GH3 cells and probably indirect ang IV action via its degradation product, ang 5–8. Both ERK1/2 and p38 MAPK pathways appear to be essential for the growth-regulatory effects of angiotensin peptides in GH3 cell culture. ERK1/2 participate in the antiproliferative effect of ang II, whereas p38 MAPK appears to mediate the inhibitory actions of both ang II and ang 5–8 upon cellular survival. The mechanism through which ang 5–8 inhibits proliferation in the GH3 cells remains to be clarified.
